# Microwaves from mobile phone induce reactive oxygen species but not DNA damage, preleukemic fusion genes and apoptosis in hematopoietic stem/progenitor cells

**DOI:** 10.1038/s41598-019-52389-x

**Published:** 2019-11-07

**Authors:** Matus Durdik, Pavol Kosik, Eva Markova, Alexandra Somsedikova, Beata Gajdosechova, Ekaterina Nikitina, Eva Horvathova, Katarina Kozics, Devra Davis, Igor Belyaev

**Affiliations:** 1 Deparment of Radiobiology, Cancer Research Institute, Biomedical Research Center, University Science Park for Biomedicine, Slovak Academy of Sciences, Bratislava, Slovak Republic; 2 Deparment of Genetics, Cancer Research Institute, Biomedical Research Center, University Science Park for Biomedicine, Slovak Academy of Sciences, Bratislava, Slovak Republic; 3 Department of Oncovirology, Cancer Research Institute, Tomsk National Research Medical Center, Russian Academy of Sciences, Tomsk, Russia; 4 The Hebrew University Hadassah School of Medicine, and Environmental Health Trust, Washington, USA

**Keywords:** Apoptosis, Double-strand DNA breaks, Risk factors

## Abstract

Exposure to electromagnetic fields (EMF) has been associated with the increased risk of childhood leukemia, which arises from mutations induced within hematopoietic stem cells often through preleukemic fusion genes (PFG). In this study we investigated whether exposure to microwaves (MW) emitted by mobile phones could induce various biochemical markers of cellular damage including reactive oxygen species (ROS), DNA single and double strand breaks, PFG, and apoptosis in umbilical cord blood (UCB) cells including CD34+ hematopoietic stem/progenitor cells. UCB cells were exposed to MW pulsed signals from GSM900/UMTS test-mobile phone and ROS, apoptosis, DNA damage, and PFG were analyzed using flow cytometry, automated fluorescent microscopy, imaging flow cytometry, comet assay, and RT-qPCR. In general, no persisting difference in DNA damage, PFG and apoptosis between exposed and sham-exposed samples was detected. However, we found increased ROS level after 1 h of UMTS exposure that was not evident 3 h post-exposure. We also found that the level of ROS rise with the higher degree of cellular differentiation. Our data show that UCB cells exposed to pulsed MW developed transient increase in ROS that did not result in sustained DNA damage and apoptosis.

## Introduction

In recent times, worldwide exposure to microwaves (MW) emitted by growing number of wireless devices had increased rapidly^1,2^. In 2011, MW were classified as a possible carcinogen (2B) by the International Agency for Research on Cancer (IARC)^3,4^. Despite this classification the health effects of MW exposure in terms of cancer or other diseases are still disputed. Safety limits set by the International Commission on Non-Ionizing Protection (ICNIRP) took into account only acute thermal effects characterized by the specific absorption rate (SAR) and did not develop any specific standards for children and pregnant women, or other potentially sensitive populations^5^. Based on observed non-thermal effects of chronic exposure to MW characterized in by physical variables as frequency, polarization and modulation^6^, alternative recommendations on safety limits took into account evidence for non-thermal effects and especially recommend reducing exposures to children^7,8^.

Results of case-control epidemiological studies have consistently showed higher risk of a glioma and acoustic neuroma in people using mobile phone for more than seven years^9–13^. The mechanisms responsible for carcinogenic effects of MW are still not fully understood and results of experimental studies seem to be inconsistent due to differences in an experimental design and number of exposure parameters. Despite these differences between studies a significant number of publications reported an increased level of reactive oxygen species (ROS) after MW exposure^14–19^. On the other hand, there were few studies where no effect of MW exposure on ROS level was described^20,21^. Recent reviews revealed that 90% of all studies analyzing the ROS after MW exposure have reported ROS induction in different cell types^22,23^. The increased level of ROS is often associated with oxidative DNA damage^15,17,24^. DNA double strand breaks (DSB), the most detrimental type of DNA damage, could be analyzed by immunolabeling and quantification of discrete DNA repair foci either by fluorescent microscopy^25,26^ or imaging flow cytometry^27,28^. DNA repair foci are formed by proteins such as γH2AX and 53BP1, which take part in DNA damage response and DSB repair^29,30^. Analysis of DSB after MW exposure revealed different data ranging from increased level of DSB^31,32^, no effect^21,24,33^ up to inhibition of DSB repair^34–36^. At the same time, number of studies described increased level of DSB and single strand breaks (SSB) in rat and mouse brain cells using comet assay^37–40^. The same inconsistence, ranging from the effect^31,41^ of MW exposure to no such effect^21,42,43^, is present in studies on apoptosis after MW exposure. The main source of this inconsistence could be high number of different biological (i.e. cell type, sex, cultivation medium, concentration of oxygen, presence of antioxidants) and physical variables (i.e. frequency, polarization, modulation, background magnetic fields) that may account for the presence or lack of the biological effect of MW exposure^6,44^.

Although childhood leukemia is relatively rare disease, it remains, along with brain neoplasms, the most common childhood cancer. It is commonly accepted that childhood leukemia arises from hematopoietic stem/progenitor cells (HSPC) by induction of mutations and formation of preleukemic fusion genes (PFG) that are considered as a first hit^45^ and can be detected in umbilical cord blood (UCB)^46,47^. To the best of our knowledge, induction of PFG by the mobile phone MW has not been studied yet. HSPC populations are usually distinguished by immunophenotyping using cluster of differentiation (CD) markers, specifically CD34 and CD38 proteins. Population enriched for hematopoietic stem cells (HSC) is CD34+ CD38−, while progenitor cells are characterized by the CD34+ CD38+ immunophenotype^48^. In this study we analyzed DNA damage, apoptosis, ROS, and PFG induction in UCB hematopoietic cells including CD34+ CD38−/CD38+ HSPC after exposure to MW from widely spread GSM900 and UMTS mobile phones.

## Results

### Reactive oxygen species

**Figure 1:**
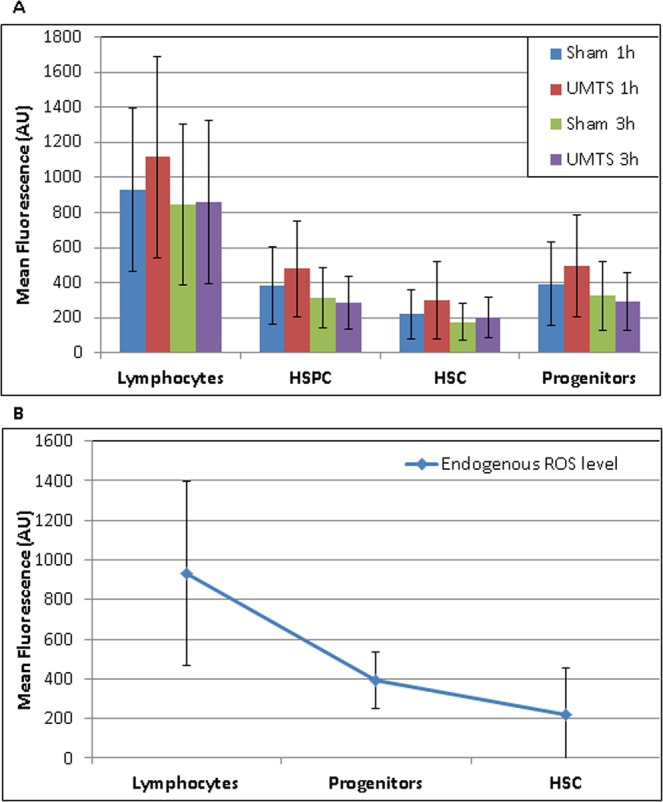
Figure shows: (**A**) level of ROS in different UCB subpopulations (lymphocytes, CD34+ HSPC, CD34+ 38− HSC and CD34+ 38+ progenitors) after exposure and sham-exposure with UMTS MW; (**B**) endogenous level of ROS in lymphocytes, progenitors and HSC. Mean value and SD from 4–6 independent experiments is shown in each data point.

We exposed UCB mononuclear cells (MNC) to UMTS MW with the frequency of 1947.4 MHz and the SAR of 40 mW/kg for 1 or 3 h and analyzed ROS immediately after exposure in different subpopulations of hematopoietic cells, specifically in CD45+ lymphocytes, CD34+ CD38− HSC, and CD34+ 38+ progenitor cells (Fig. 1A). In parallel experiments, we observed very rapid induction of ROS with mean fluorescence intensity up to 60000 arbitrary units after treatment with TBHP (Supplementary Fig. 1). Analysis of variance (ANOVA) showed that the ROS level after 1- and 3-h exposure is dependent both on exposure time (p = 0.001) and cell type (p = 0.0000001). Next we analyzed samples exposed for 1 and 3 h separately. We found statistically significant induction of ROS after 1 h exposure almost in all studied populations, namely: 22% increase in CD45 cells (p = 0.04) and 27% increase in CD34+ HSPC (p = 0.03). Further analysis of CD34+ cells showed 28% increase in progenitors (p = 0.05), while no significant increase in HSC (p = 0.1). However, no such effect has been detected after 3-h exposure. In order to verify whether the loss of effect after 3 h may be caused by a change in the oxygen concentration in the medium with cells we measured this concentration. The measurements revealed a decrease in the oxygen concentration during incubation (1 h, cO2 = 7.48 µg/ml; 3 h, cO2 = 7.1 µg/ml). Thus, the result may well be due to the incubation conditions rather than adaptation to MW exposure. We conclude that MW exposure at selected conditions may increase the ROS in different types of hematopoietic UCB cells although the observed increase was in the limits of physiological changes (Fig. 1A). We also found that endogenous levels of ROS differed between cell populations (Fig. 1B). HSC had significantly lower level of ROS than both progenitors (p = 0.015) and lymphocytes (p = 0.0004). This data correlated well with the lower level of endogenous DNA damage in HSC as previously reported^46,49^. In turn, progenitors had also significantly lower endogenous level of ROS than lymphocytes (p = 0.002). Thus, endogenous level of ROS rose with the higher level of differentiation (HSC < progenitors < lymphocytes).

### DNA repair foci

**Figure 2:**
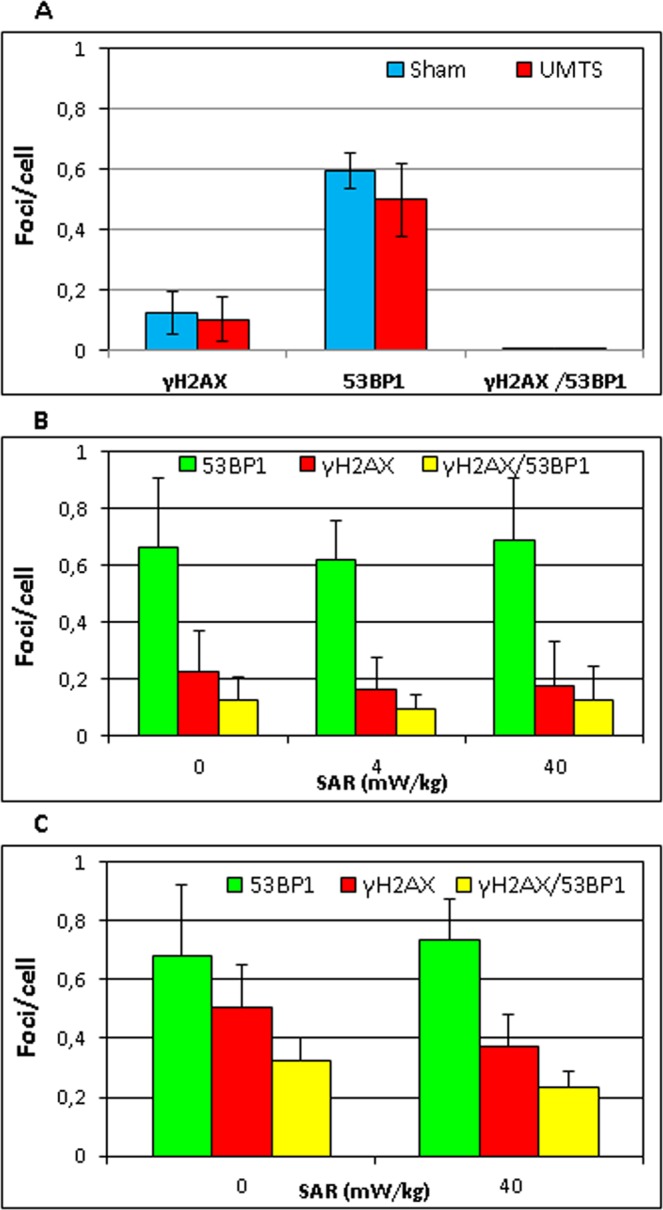
Mean number of γH2AX/53BP1 per cell in UCB MNC after: (**A**) 3-h exposure to UMTS MW, (**B**) short exposure (1–4 h) to GSM MW, and (**C**) prolonged exposure (17 h) to GSM MW. Data are from 7 (**B**) or 3 (**A**,**C**) experiments.

To asses possible causative relationship between increased ROS and DNA damage we studied whether UMTS MW induced DSB by quantifying them using γH2AX/53BP1 foci. Effect of UMTS MW with the SAR of 40 mW/kg was studied by imaging flow cytometry in UCB MNC after 3 h of MW exposure, since formation of foci require some time after induction of DNA damage and these foci persist for at least 2 h after induction^49^. However, imaging flow cytometry did not reveal any difference between UMTS MW exposed and sham exposed MNC UCB in the level of γH2AX (p = 0.75), 53BP1 (p = 0.33), and γH2AX/53BP1 foci (p = 0.52) (Fig. 2A).

Effect of GSM MW exposure was studied using the Metafer microscopy system, which provides for higher resolution than imaging flow cytometry^27^. We performed short (1–4 h) and long (17 h) exposures with GSM MW with the frequency 915 MHz at different SAR (4 and 40 mW/kg). In the group of short exposures, the level of γH2AX, 53BP1, and γH2AX/53BP1 co-localizing foci was not affected by the exposure time as analyzed by the Univariate ANOVA. Thus, we pooled the data from the short exposures for further analysis. Short exposure of UCB cells to GSM MW did not induce DSB at both SAR values as was found by the enumeration of γH2AX (SAR 4 mW/kg, p = 0.34; SAR 40 mW/kg, p = 0.5), 53BP1 (SAR 4 mW/kg, p = 0.6; SAR 40 mW/kg, p = 0.81), and γH2AX/53BP1 foci (SAR 4 mW/kg, p = 0.34; SAR 40 mW/kg, p = 0.95) (Fig. 2B). Prolonged exposure to GSM MW at the SAR 40 mW/kg also did not induce DSB as revealed by analysis of γH2AX (p = 0.13), 53BP1 (p = 0.43), and γH2AX/53BP1 foci (p = 0.31) (Fig. 2C). Positive control, γ-irradiation with 2 Gy, showed rapid induction of γH2AX/53BP1 foci 30 minutes after irradiation (mean values: 20 γH2AX foci/cell,12.7 53BP1foci/cell, and: 10.8 γH2AX/53BP1 co-localized foci/cell) (see Supplementary Fig. 2).

### Comet assay

**Figure 3:**
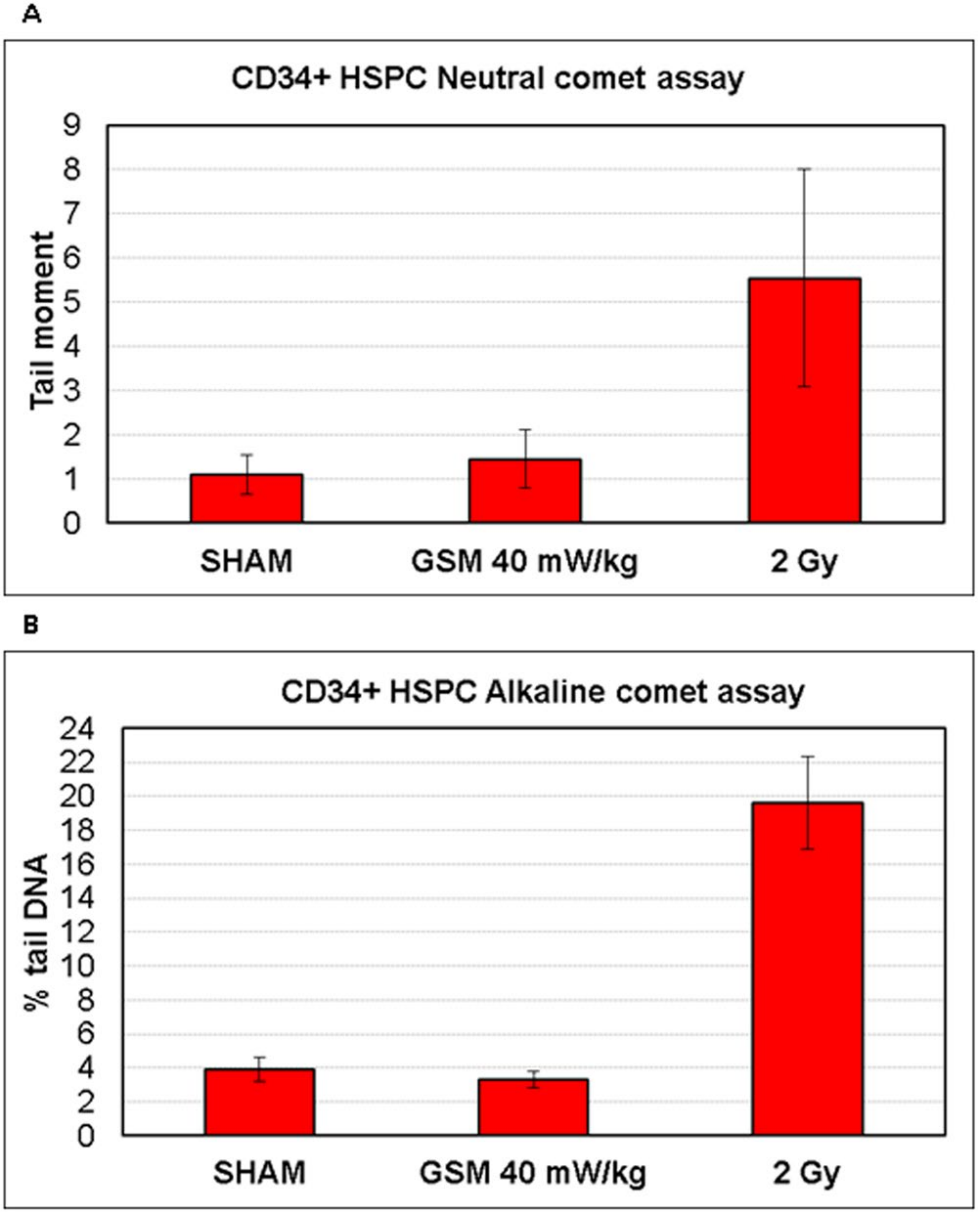
DNA damage after GSM MW exposure and 2 Gy of γ-rays analyzed by comet assay in CD34+ HSPC cells. Mean tail moment (**A**) and % of tail DNA (**B**) along with SD from two independent experiments (triplicated in each) is shown.

DNA damage after 1 h of GSM MW exposure was also assessed in CD34+ HSPC using the neutral and alkaline comet assay. As a positive control we irradiated cells with 2 Gy of γ-rays. Cells were taken for comet analysis immediately after exposure. We did not see any significant effect GSM MW exposure with SAR 40 mW/kg on the level of DNA damage using either neutral or alkaline comet assay (Fig. 3A,B). In contrast, after irradiation with 2 Gy we have found a highly significant induction of DNA damage using neutral and alkaline comet assay (t-test, p = 0.002 and 0.000002, respectively). These results are in line with the data from analysis of DNA repair foci.

### Apoptosis

**Figure 4:**
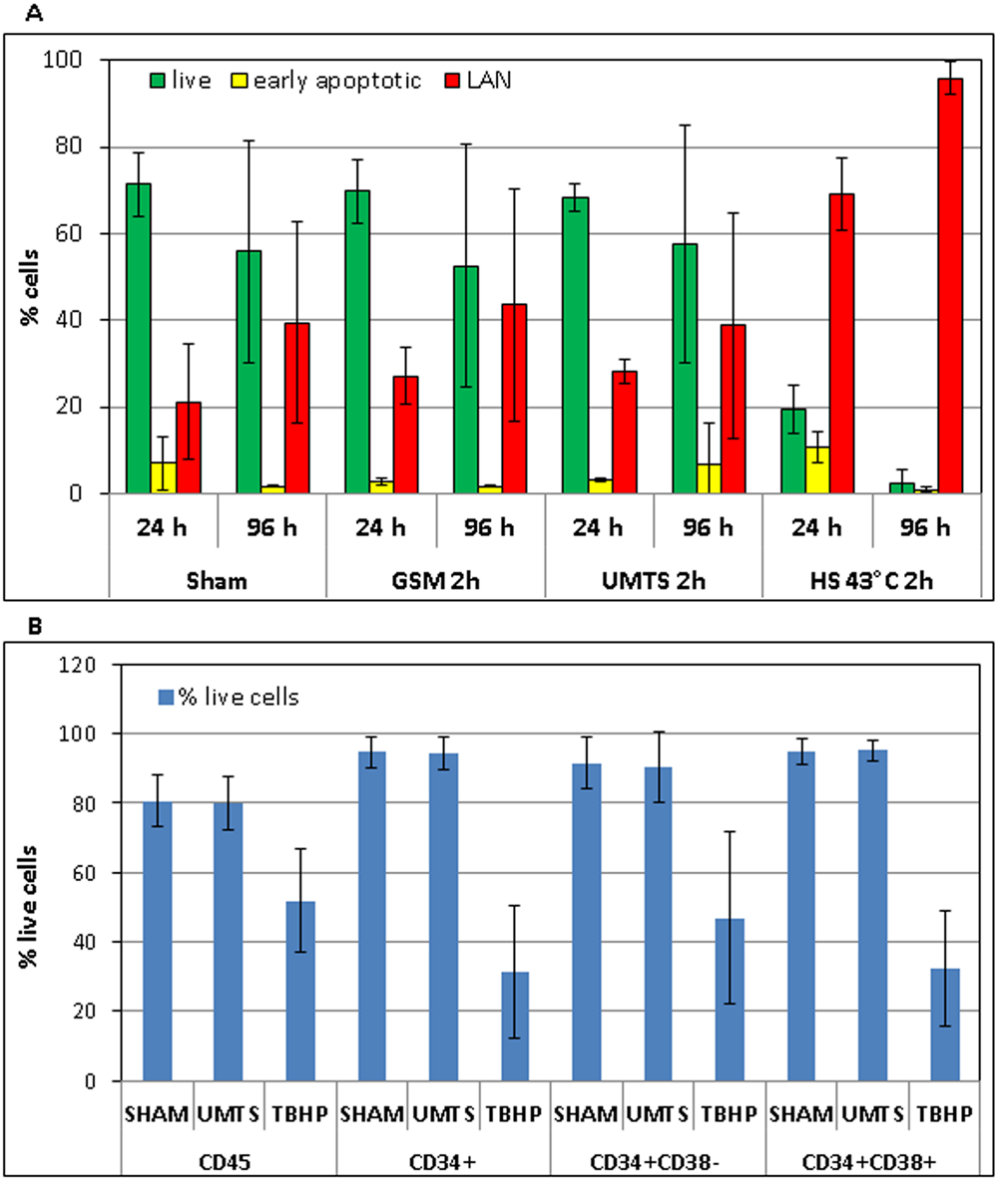
(**A**) Apoptosis in CD45+ UCB cells were analyzed 24 and 96 h after 2 h exposure to GSM and UMTS MW, sham-exposure and hypertermia by the Annexin/PI assay. Data from four independent experiments are shown. (**B**) Percentage of live cells in different populations of UCB cells (CD45+, CD34+ HSPC, CD34+ 38+ progenitors, and CD34+ 38− HSC) were analyzed immediately after 1 h exposure/sham-exposure with UMTS MW or treatment with 50 mM solution of tert-butyl hydrogen peroxide (TBHP). Data from 5 to 6 independent experiments are shown in each data point.

To verify whether increase in the level of ROS induced by MW exposure could lead to an apoptotic response we performed Annexin-V/PI assay using standard flow cytometry. We measured percentage of live, early apoptotic and late apoptotic/necrotic (LAN) cells 24 and 96 h after 2 h exposure with UMTS MW at the SAR of 37 mW/kg, the frequency of 1947.4 MHz and also after 2-h exposure with GSM MW at the SAR of 40 mW/kg, the frequency of 915 MHz. Hyperthermia for 2 h at 43 °C was used as a positive control. Hyperthermia induced apoptosis significantly (p = 0.000001), but we did not observe any change in cell survival after exposure to both UMTS (p = 0.55) and GSM MW (p = 0.45) either 24 or 96 h after exposure (Fig. 4A). We conclude that the UMTS and GSM MW did not induce apoptosis in the UCB MNC at our experimental settings.

Along with measuring formation of ROS we have also analyzed percentage of dead cells (7-AAD+) by flow cytometry. Amount of dead cells supported the results obtained by the Annexin-V/PI analysis, as we did not see any difference between the sham exposed and UMTS exposed samples in either CD45+ or CD34+ HSPC, CD34+ 38+ progenitors and CD34+ 38− HSC (p = 0.9; 0.92; 0.84; 0.88 respectively). At the same time, we observed lower cell viability in all studied cell populations (CD45+, CD34+, progenitors, and HSC) after treatment with 200 µM solution of TBHP, that we used as a positive control for ROS induction (p = 0.002; 0.00002; 0.005; 0.00004 respectively). In line with our previous studies^46,49^ we found higher viability in CD34+ HSPC compared to their differentiated progeny, CD45 cells (p = 0.003). There was no statistically significant difference in viability between progenitors and HSC (p = 0.4) (Fig. 4B).

### Preleukemic fusion genes

We also examined whether exposure of UCB MNC to GSM and UMTS MW could induce translocations resulting in preleukemic fusion genes (PFG) formation. Using RT-qPCR, we have screened for TEL-AML1 and BCR-ABL fusion genes, which are most abundant in acute lymphoblastic leukemia (ALL). TEL-AML1 fusion gene was analyzed in 3 independent experiments, where the cells were exposed to GSM MW for 10 h in average with SAR values 4 mW/kg and another 3 with the SAR 40 mW/kg. Due to limited number of cells, the only one exposure condition was applied to UCB MNC from each proband. We have found TEL-AML1 gene fusion in one exposed sample in one of the experiments with the SAR of 4 mW/kg. In other experiments we found no instance of fusion genes (Table 1).We have also analyzed TEL-AML1 fusion gene after exposure to UMTS MW but only observed positive results in the sham-exposed sample (Table 1). BCR-ABL fusion gene was analyzed in two experiments with UMTS MW exposure and one experiment with GSM MW exposure with SAR 40 mW/kg. All exposed and sham-exposed samples were negative for the expression of BCR-ABL fusion gene (Table 1). The number of neither TEL-AML1 nor BCR-ABL fusion gene transcripts was not higher than 5 copies per 100 000 cells. This highlights the fact that analysis of low-copy numbers of PFG is at the border of RT-qPCR sensitivity as was shown in our previous studies^46,50^.

**Table 1:** Table shows PFG in UCB MNC after exposure to GSM and UMTS MW as analyzed by the RT-qPCR.

TEL-AML1	P51	P139	P141	P144	P150	P9	P378
SHAM	0/3	0/3	0/3	1/3	0/3	0/3	0/3
GSM 4 mW/kg	1/3					0/3	
GSM 40 mW/kg		0/3	0/3				0/3
UMTS				0/3	0/3		
**BCR-ABL**	P190	P150	P214				
SHAM	0/3	0/3	0/3				
GSM 40 mW/kg			0/3				
UMTS	0/3	0/3					

**Figure 5:**
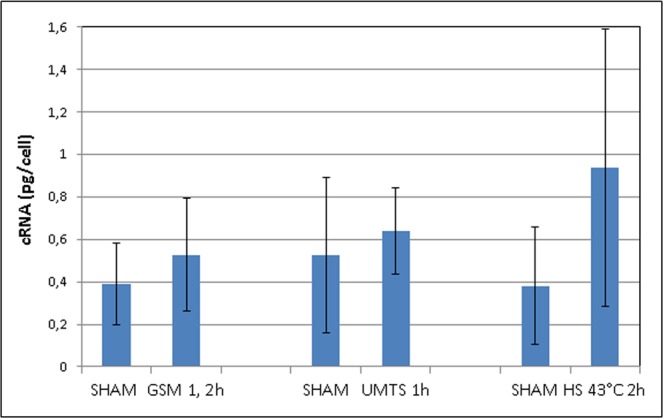
Per-cell RNA amount (pg/cell) after MW exposure (GSM, UMTS), sham exposure, and hyperthermia. Data are shown from 16 experiments for GSM exposure, 9 experiments for UMTS exposure, and 3 experiments for hyperthermia.

Finally we evaluated the per-cell content (Fig. 5) of RNA after exposure to GSM and UMTS microwaves and also with hyperthermia. We did not observe any significant differences in the content of RNA per cell, although a trend to a higher value was present at all exposure conditions suggesting higher transcription activity in MW and hyperthermia exposed cells (Fig. 5). Thus, temperature may be a co-factor that heightens *in vitro* sensitivity to MW.

## Discussion

Herein, we aimed to study ROS, DNA damage, apoptosis, and PFG after exposure of hematopoietic cells to MW from GSM and UMTS mobile phones at intensities well below any thermal effects. We analyzed both matured lymphocytes and HSPC, which are commonly considered as a cellular target for origination of leukemia^51^. Cells derived from UCB were used as a model system, since UCB contains significantly higher percentage of HSPC compared to peripheral blood. We assessed DSB by analyzing γH2AX/53BP1 DNA repair foci after GSM MW exposure at the frequency of 915 MHz and UMTS MW at the frequency of 1947.4 MHz. At our experimental settings, we did not find any change in the level of foci. In multiple parallel experiments with ionizing radiation, we defined sensitivity of our DNA repair focus assay being sensitive to about 0.2 DSB/cells^27,49,52^. We concluded that MW induced no DSB within the limits of the DNA repair focus assay’s sensitivity. These results are in contrast with previous studies, where decrease in the level of γH2AX/53BP1 foci paired with higher order of chromatin condensation was found in human peripheral blood lymphocytes (PBL) after exposure to GSM MW under similar conditions^34–36,42^. However, there were at least three differences between the current and the aforementioned previous studies. First, we used frozen UCB cells while freshly isolated PBL have previously been used. Thawing of frozen cells might affect their sensitivity to MW. Second, we fixed cells immediately after exposure although the PBL were kept for 1 h on ice after exposure. This cold treatment might facilitate response to MW. Third, background ELF and static magnetic fields were somewhat different between previous and present studies possibly underlying eventual inconsistency. Indeed, there was higher level of background ELF, 2 µT, during UMTS exposures in present study compared to 0.2 µT in study by Markova *et al*.^35^. The values of stray SMF/ELF was 37 µT/2 µT during exposure to GSM MW in the present study while 18 µT/200 nT was during MW exposures in Markova’s study. Of note, change in stray SMF/ELF by 20/2 µT was reported to vanish non-thermal effects of MW^6^. Contrary to the results of the present study and studies by Markova *et al*. and Belyaev *et al*., increased level of DSB and SSB was reported by others using comet assay^37–40^. This variability in outcome is likely accounted for strong dependence of MW effects on number of biological and physical parameters, which differed significantly between studies^6^. In line with our DNA repair focus data, we did not see any effects of GSM MW on DNA damage as analyzed by comet assay. Neither neutral nor alkaline comets were changed by the MW exposure. On the other hand Glaser *et al*. who studied DNA damage in CD34+ HSPC using alkaline comet assay have observed decreased comet tails after exposure to GSM MW^21^. While these data were interpreted as decreased DNA damage, the affected tail moment may be caused by other reasons changing migration of intact DNA loops. Direct prove that both increased and decreased comets as measured with neutral comet assay may not be dealt with DNA damage was provided by different research groups^53,54^. Notably, it has been shown that increased or decreased neutral comets were caused by decondensation or condensation, respectively, of intact DNA loops. The increased tails measured with alkaline comet assay may be caused by increased number of open replicons or transcription units due to their inhibition prolongation but not by DNA damage.

A number of studies had described an impact of various biological^55–57^ and physical^6,44^ parameters on the presence of MW exposure effect. Difference in these parameters (incubation on ice, usage of frozen cells, SMF, ELF etc.) could mirror into different effect of MW exposure observed in present and previous publications. Insufficient description of experimental parameters is often the case that could lead to very low reproducibility of the MW effects. To the best of our knowledge, effect of MW exposure on DNA damage was shown in about 50% of studies published so far while there was no effect found in other studies. As discussed above, this inconsistency could be caused by the presence of DNA –damaging effect only at specific parameters like the type of signal and exposure conditions^3,6^.

We also studied apoptosis and ROS after exposure to GSM and UMTS MW in different subpopulation of UCB cells. We did not observe any induction of apoptosis neither 24 nor 96 h after exposure to MW, but we found very high induction of apoptosis after hyperthermia. Our results are in line with the results by some others. For example, Liu *et al*. that also did not observed any differences in apoptosis after exposure of mouse cells with GSM MW at 1800 MHz^43^. Also Belyaev *et al*. did not find out any difference in apoptosis after GSM MW exposure^42^. Glaser *et al*. who studied apoptosis in leukemic cell line HL-60 and CD34+ HSPC cultured with growth factors did not find any induction of apoptosis by none of the GSM (900 MHz), UMTS (1950 MHz) and LTE (2535 MHz) MW signals^21^. On the other hand, other studies revealed induction of apoptosis after MW exposure^31,58^ and also induction of pro-apoptotic proteins^58^. Despite the fact that at our experimental settings we did not observe any effect of MW exposure on DSB and apoptosis we found induction of ROS after 1-h UMTS exposure that is in line with a number of other studies^14–19^. In contrast, Glaser *et al*. have not observed induction of ROS in CD34+ HSPC by none of the GSM, UMTS, and LTE MW signals. These authors have quantified the ROS level after 4 and 24 h of exposure, while we have detected increased level of ROS after 1-h exposure. Of note, we found ROS induction in all studied cell populations (lymphocytes, CD34+ 38+ progenitors, and CD34+ 38− HSC). Increase in the ROS level was approximately 30% in average with strong interindividual differences (2–60% of increase), although this increase was present in every experiment. Other authors observed similar^43^ or even higher^58^ level of ROS induction after MW exposure. The revealed change in ROS were time dependent as far as we did not detect any effect of MW exposure on the level of ROS at 3 h. Similar time dependent MW effects on ROS were described by Xing *et al*. who reported ROS induction in glioblastoma cells after 3-h exposure, while this effect decreased after 6 h and vanished after 12, 24 and 48 h of exposure^58^. Difference in time kinetics of the MW-induced ROS between ours and Xing’s study could be accounted for different cell types and exposure conditions. Taking into account that exposure to MW is basically life long, even slight increase in ROS observed after short-term exposures may be of potential biological relevance for carcinogenesis if exposure is lifelong^3^. In our study, for the first time, we compared endogenous levels of ROS in HSC and progenitors with lymphocytes. Notably, the endogenous level of ROS rose with the higher degree of cell differentiation showing the lowest level in HSC (CD34+ 38−), moderate in progenitor cells (CD34+ 38+), and the highest in lymphocytes (CD45+ 34−). The most probably these differences in ROS are related to the lower mitochondrial metabolism oxygen consumption activity in quiescent stem/progenitor cells^59^. The presented results are in line with the literature data that described low mitochondrial oxygen consumption and cell metabolism in human CD34+ HSPC^59–61^.

In present study, for the first time, we analyzed induction of PFG in hematopoietic cells exposed to MW. We have chosen TEL-AML1 and BCR-ABL, which are most abundant preleukemic fusion genes for ALL. While we detected induction of TEL-AML1 fusion after MW exposure in one experiment, in other one we found TEL-AML1 in sham exposed, but not in MW exposed sample. PFG detected in UCB are usually present in the very low copy numbers, arguably on the limit of RT-qPCR sensitivity^46^. Thus, number of PFG copies detected in this study was not higher than 5 copies per 100 000 cells, that is in line with our previous studies^46,50^. From obtained results we are not able to draw any conclusion, whether exposure to mobile phone MW could have any role in forming PFG and higher risk of childhood leukemia. Finally, we conclude that MW exposure under specific condition of exposure induce ROS reversibly in various hematopoietic cell types including HSC. However, this induction was not followed by increased DNA damage, apoptosis or formation of preleukemic fusion genes.

## Materials and Methods

### Chemicals

Reagent grade chemicals were obtained from Sigma-Aldrich (St. Louis, Missouri, USA) and Life technologies (Carlsbad, California, USA).

### Ethical considerations

This study has been approved by the Ethics Committee of Children’s Hospital in Bratislava and all experiments and methods were performed in line with relevant guidelines. All UCB samples were provided with an informed consent from a parent or legal guardian for study participation.

### Cells

Mononuclear cells (MNC) were isolated from UCB as previously described^52^ by Dr M. Kubes (Eurocord, Slovakia), and cryopreserved in liquid nitrogen. Before experiments, the cells were thawed in water bath and transferred to RPMI medium. Adherent monocytes were removed by the 2 h incubation of cells in RPMI medium supplemented with 10% FBS and 100 IU/ml penicillin, 100 μg/ml streptomycin at 37 °C in a 5% CO_2_-incubator. After incubation the most of the MNC population were lymphocytes and about 1% accounted for CD34+ HSPC. Viability of remaining MNC was higher than 95% as defined by the Trypan blue exclusion assay.

### Cell exposure

Cells were exposed to the mobile phone emitted MW using either GSM 900 test mobile phone (model GF337; Ericsson, Stockholm, Sweden) or UMTS test mobile phone (model 6650, Nokia, Helsinki, Finland), output power being the same 0.25 W, under well controlled conditions essentially as previously described^34,35,42,62^. Briefly, exposure and sham exposure of cells with GSM 900 phone were performed simultaneously at 37 °C in a humidified CO_2_ incubator (Heracell™ 150i, Thermo Fischer Scientific, Waltham, Massachusetts, United States) in two specially designed identical transverse electromagnetic transmission line (TEM) cells connected to output of the mobile phone by coaxial cable. There are 124 different channels/frequencies, which are used in GSM900 mobile communication. They differ by 0.2 MHz in the frequency range between 890.2 MHz and 914.8 MHz. GSM signal is pulsed by 577 μs pulses (time slots), 2 W power in pulse, with the waiting time of 4039 μs (7 time slots) between pulses. Our test-mobile phone was programmed to use a pre-set frequency and regulate output power in pulses in the range of 0.02–2 W (13–33 dBm). For GSM 900 exposure we used channel 124 with the frequency of 915 MHz and two different SAR values of 4 and 40 mW/kg as defined by the finite different time domain (FDTD) method. The signal included standard GSM modulation, Gaussian Minimum Shift Keying (GMSK). Discontinuous transmission mode was off during exposures. Static magnetic field (SMF) was 37 μT at the places of real and sham GSM exposures as measured by a TM75-41 magnetometer (Izmiran, Fryazino, Russia). Background extremely low frequency magnetic fields (ELF) did not exceed 2 μT root mean square (rms), as measured with a three-dimensional TM-192 microteslameter (Tenmars, Taipei, Taiwan). UMTS exposure (1947.4 MHz middle channel, 5 MHz wide band, and SAR of 40 mW/kg) and sham exposure was performed simultaneously in two identical TEM-cells in other incubator at 37 °C. The UMTS signal included its standard modulation, Quadrature Phase Shift Keying (QPSK). SMF at the places of real and sham UMTS exposures was 65 μT. Background ELF magnetic field was not more than 2 μT rms at the places of real and sham UMTS exposures. Voice modulation was not applied either in GSM or in UMTS exposures. In our TEM cells, the power loss controlled with a power meter (Bird 43, USA) or a power meter (Hewlett-Packard 435 A, USA) did not exceed 1.2% and that could not cause any temperature rise. Our TEM-cells were well ventilated through the special holes in the wooden cages. Temperature was measured in the MW-exposed samples before, during and after exposure with a precision of 0.1. No changes in temperature were induced during exposure. Taking into account all possible uncertainties, the SAR values at all locations within exposed samples were always well below thermal effects.

### Reactive oxygen species

ROS were analyzed using Cell ROX Green kit (Life technologies, New York, USA) according to manufacturer’s instructions. For positive control, cells were treated with Tert-butyl hydrogen peroxide (TBHP) (working concentration 200 µM). Briefly, after exposure or sham exposure, 1 million cells in 500 µl were taken for ROS measurement and 2 µl of 2.5 mM Cell ROX solution was added directly to the cells in RPMI and incubated for 45 min in CO_2_ incubator with closed caps. After 20 min of incubation antibodies against surface markers were added, specifically, CD45-V450 conjugate (BD biosciences, San Jose, California, USA), CD34-APC conjugate (Myltenyi Biotec, Bergisch Gladbach, Germany) specific for HSPC, CD38-PeCy7 (BD biosciences) conjugate to distinguish population that is enriched for either HSC (CD34+ 38−) or progenitors (CD34+ 38+) and 3 µl of 7-AAD for dead cells staining. Samples were then incubated for another 25 minutes in CO_2_ incubator and subsequently analyzed by flow cytometer (BD FACS Canto II). Data were analyzed via BD FACS Diva software. Compensation matrix was created by the compensation wizard in the FACS Diva software after acquisition of single color stained samples and unstained control.

### DNA repair foci

DNA repair foci were analyzed by imaging flow cytometry and automated fluorescent microscopy as previously described^27,52^. Briefly, 3–5 million of cells were fixed immediately after exposure and immunostained with the primary polyclonal rabbit 53BP1 antibody (Novus biologicals, Cambridge, United Kingdom). As a positive control cells were irradiated with 2 Gy of γ-rays at a dose rate of 0.35 Gy/min using a *THERATRON*^*®*^ *Elite 80* (MDS Nordion, Ottawa, Canada). After 1 h incubation secondary antibody Alexa Fluor 488 anti-rabbit 1:200 (Life technologies) and monoclonal mouse γH2AX BV-421 conjugate in dilution 1:10 were added and cells were incubated for 1 h at room temperature in the dark. Subsequently, the cells were washed in cold PBS and stained with 2 μl of 7-AAD for DNA staining (BD biosciences). From each sample, at least 15000 cells were captured using the ImageStream^X-100^ (Amnis Inc., Seattle, Washington, USA) with 60x objective and the extension depth of field 1 (EDF1) to gain the best possible resolution. Three lasers, 405, 488, 785 nm and CCD camera were used to analyze γH2AX, 53BP1, DNA, granularity, and cell morphology, respectively. Images of cells were acquired at the rate of 50–200 cell/s. Using the IDEAS software, image compensation was performed and DNA repair foci were enumerated in appropriate cells essentially as previously described^27^.

For automated fluorescent microscopy cells were cytospun on microscopic cytoslides immediately after exposure (ThermoShandon, Pittsburgh, Pennsylvania, USA) before being fixed and immunostained as previously described^26,27,52^. A primary antibody mix consisting of 53BP1 polyclonal/rabbit antibody at 1:800 dilution and γH2AX monoclonal/mouse antibody (both antibodies from Novus biologicals) at 1:400 dilution was used. The secondary antibody mix consisted of Alexa Fluor 488 IgG (H + L) anti-rabbit, 1:200, and Alexa Fluor 555 IgG (H + L) antimouse, 1:200 (Life Technologies). Upon immunostaining, cover slips (Menzel-Gläser, Germany) were mounted on microscopic cytoslides with a VECTASHIELD mounting medium (Vector Laboratories, Peterborough, United Kingdom) and sealed using a translucent nail polish. The slides were scanned by the METAFER Slide Scanning System (MetaSystems, Altlussheim, Germany). Firstly, optical sections through the nuclei were captured from 10 sections at 0.7 µm intervals and the final images were obtained by the projection of the individual sections with a Zeiss Axio Imager Z1 microscope (Zeiss Microscopy, Jena, Germany) using the 63x objective. DNA repair foci were enumerated by custom made classifier in at least 150 cells and data from two microscopic slides were pooled together.

### Comet assay

DNA damage response was detected in parallel by alkaline^63^ and neutral single-cell gel electrophoresis (SCGE)^64^ also called comet assay. Briefly, suspensions of the probands cells in 0.75% LMP agarose dissolved in PBS was spread onto microscopic slides pre-coated with 1% NMP agarose. The cells were then lysed for 1 h at 4 °C in a buffer consisting of 2.5 M NaCl, 0.1 M Na_2_EDTA, 10 mM Tris-HCl and 1% Triton X-100, pH = 10. After the lysis, the slides were placed in an electrophoresis box for DNA unwinding for 40 min in the electrophoretic solution (0.3 M NaOH and 1 mM Na_2_EDTA, pH > 13). Electrophoresis was conducted at temperature of 4 °C for 30 min at electric field strength 0.73 V/cm. The slides were then neutralized in 0.4 M Tris-HCl, drained, stained with 5 µg/ml ethidium bromide (EtBr) and covered with cover slips. The neutral assay was performed at pH = 9 essentially according to the same procedure as the alkaline version except the composition, pH value and duration of individual steps. In the neutral version, lysis was performed at the alkaline lysis solution supplemented with 2% sarcosyl. Unwinding (20 min) and electrophoresis (60 min at electric field strength 0.41 V/cm) steps were carried out in a buffer consisting of 0.1 M Tris-HCl and 0.3 M sodium acetate at pH adjusted to 9 by acetic acid^64^.

At least one hundred of EtBr-stained nucleoids per exposure condition in one electrophoresis run were examined with fluorescence microscope using an automatized Metafer system. The percentage of DNA in the tail and tail moment was used as a parameter for the measurement of DNA damage in alkaline and neutral SCGE, respectively.

### Apoptosis

Cells were harvested at different time points after exposure and apoptosis was analyzed as previously described^49^. Briefly cells were spun down (100 g/10 min), washed with PBS (0.02% KCl; 0.8% NaCl; 0.29% Na2H3PO4 x 12H2O; 0.02% KH3PO4 in deionized water) and resuspended in 100 µl of the Annexin kit buffer (Roche, Basel, Switzerland). Cells were then stained with Annexin-V (Roche, Basel, Switzerland), Propidium Iodide (PI) (Roche) and anti-human CD45-V450 (BD biosciences) for white blood cell staining. Afterwards samples were incubated for 20 min in dark, washed with PBS, spun down, diluted in 200 µl of the Annexin kit buffer and analyzed by the BD FACS Canto II flow cytometer (BD biosciences). The data were obtained by the analysis with FACS Diva software and percentage of live (Annexin-V negative, PI negative), early apoptotic (Annexin-V positive, PI negative) and late apoptotic/necrotic (LAN, Annexin-V positive, PI positive) cells were assessed.

Compensations were performed on samples where LAN cells were more abundant. Single color stained tubes were acquired and compensation were generated automatically by BD FACSDiva software. As a positive control, cells were heat shocked at temperature of 43 °C for 2 h in a circulating water bath.

### RNA isolation and cDNA synthesis

RNA was isolated immediately after the end of exposure with RNAzol (Research Molecular Center, Ohio, USA) and cDNA was synthesized by reverse transcription in the standard reaction containing 1 μg of total RNA following the manufacturer’s protocol (Thermo Scientific) as previously described^50^.

### Real time quantitative PCR

RT-qPCR was performed as was described in previous reports^46,50^. The primers and probes were designed according to Gabert^65^. The plasmid standards with individual fusion genes subcloned into PCR II TOPO vector (Qiagen, Hilden, Germany). RT-qPCR was performed on a BioRad CFX96 instrument following the protocol by Gabert^65^. All precautionary measures taken against contamination have been described previously^50^. The samples were run in triplicate and regarded as positive if at least one tube was tested positive.

### Measurement of oxygen level

We also measured the concentration of oxygen (cO_2_) by the digital pH meter Multi-parameter portable meter Multi® 3420 IDS (set) with IDS Oxygen measurement cell FDO® 925 (WTW GmbH, Weilheim, Germany) in the cells under the same experimental settings (medium, cell concentration, time of incubation).

### Statistics

Analysis of normal distribution of measured end-points and of variance (ANOVA) was carried out using Statistica software (Dell software, Round Rock, Texas, United States). Comparison between treatment conditions was performed using two tailed *t*-test. The results were considered significantly different at p < 0.05

## Supplementary information

Supplementary Figure 1

Supplementary Figure 2

## Data Availability

The authors report that data could be available upon request.
