# Supplementary figures and images for: Microwaves from mobile phone induce reactive oxygen species but not DNA damage, preleukemic fusion genes and apoptosis in hematopoietic stem/progenitor cells
Source: Sci Rep. 2019 Nov 7;9:16182. doi: 10.1038/s41598-019-52389-x (PMC6838175; doi:10.1038/s41598-019-52389-x)

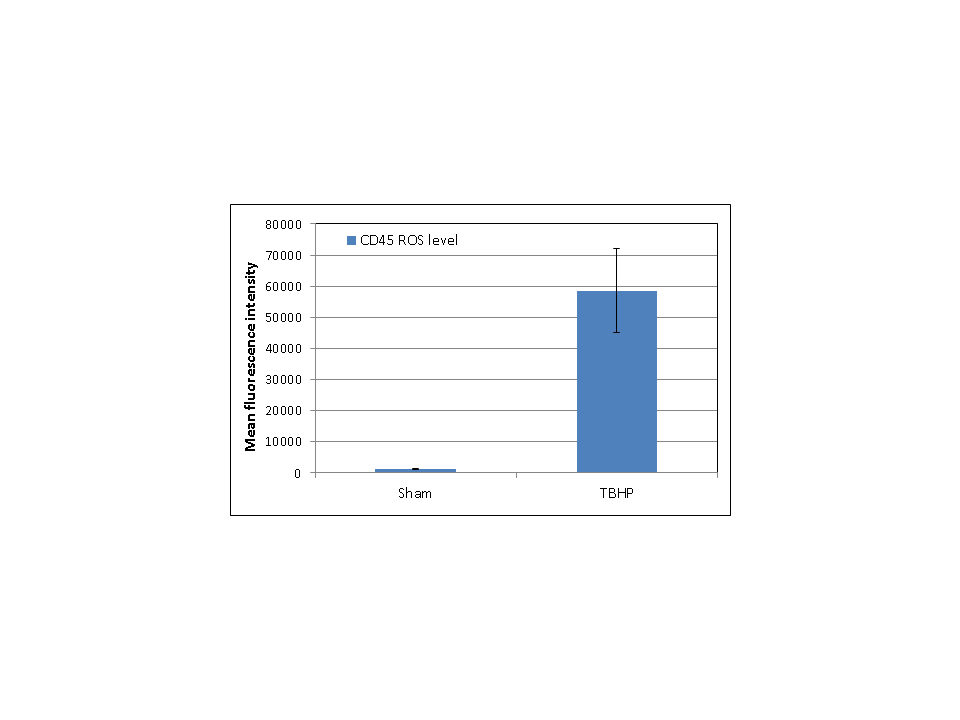

Supplement: Supplementary file 1 — Supplementary Figure 1 [file 41598_2019_52389_MOESM1_ESM.tif]

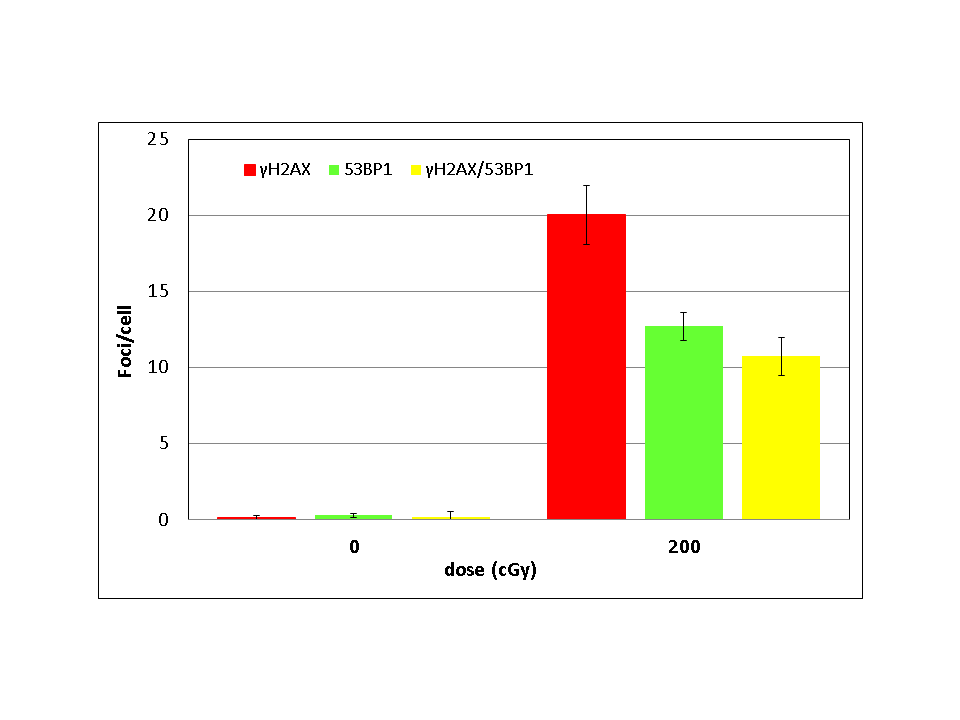

Supplement: Supplementary file 2 — Supplementary Figure 2 [file 41598_2019_52389_MOESM2_ESM.tif]
